# Evaluation of Antimicrobial Coatings on Preservation and Shelf Life of Fresh Chicken Breast Fillets Under Cold Storage

**DOI:** 10.3390/foods9091203

**Published:** 2020-09-01

**Authors:** Johanna Garavito, Diana Moncayo-Martínez, Diego A. Castellanos

**Affiliations:** 1 Post Harvest Lab, Facultad de Ciencias Agrarias, Universidad Nacional de Colombia, Bogotá 111321, Colombia; njgaravitoj@unal.edu.co (J.G.); dcmoncayom@unal.edu.co (D.M.-M.); 2 Programa de Ingeniería de Alimentos, Fundación Universitaria Agraria de Colombia, Bogotá 111166, Colombia; 3 Instituto de Ciencia y Tecnología de Alimentos, Universidad Nacional de Colombia, Bogotá 111321, Colombia

**Keywords:** food packaging, cold storage, poultry, shelf life

## Abstract

Fresh poultry products such as chicken breast are very convenient for consumption due to their availability and nutritional and sensory characteristics but they have a short shelf life (3–5 days) due to their high water activity and propensity to microbial contamination and spoilage. In this work, the characteristics of edible coatings (EC) prepared from various formulations of guar gum (GG) and isolated soy protein were evaluated. From this evaluation, and due to the guar gum EC being the most suitable, antimicrobial coatings were prepared with different proportions of GG, nisin, and oregano oil to determine its effect on shelf life and change in physicochemical and microbiological properties of chicken breast fillets under refrigerated conditions. Fresh samples were coated with a coating-forming solution based on GG and stored at 4 °C for 16 days. During this time, the change in color, firmness, acidity, pH, growth of microorganisms was determined, and sensory tests of appearance, taste, and odor were performed. GG-coated samples retained color and firmness during storage. Likewise, a decrease in weight loss was achieved in the treated samples, and the sensory attributes were preserved compared to the uncoated samples. The application of the coating considerably delayed the growth of microorganisms, increasing the product shelf life (9 days) compared to the control samples (6 days).

## 1. Introduction

Consumers currently demand fresh, natural products with added value, low cost, and low environmental impact throughout the production chain [1,2]. Fresh chicken products have become more relevant to the global consumer due to their nutritional content, relatively low cost, and the improvement in the use of meat raw material and the environmental impact of the poultry industry [2,3,4]. In Colombia, for example, the per capita consumption of chicken meat is 32.07 kg per year, ranking 28th worldwide [5]. Among the most consumed derivatives of chicken meat is the breast due to its convenience and flavor [6]. However, fresh breast meat has accelerated deterioration rates (and short shelf life) due to microbial contamination (psychrotrophic bacteria spoliation), even more so when it is conditioned and cut, which increases its surface area and susceptibility to contamination [7,8,9]. Industrially, the injection of brines and freezing treatments have been carried out in order to guarantee the preservation of this type of product cut for longer periods, resulting in negative sensory changes, loss of freshness and significant weight reduction after cooking, which the consumer detects and rejects [10].

An alternative to improve the preservation of products such as chicken breast is the use of edible coatings (EC) with which it is possible to keep the physicochemical and sensory quality at the same time for longer periods [11,12]. Edible coatings are defined as a thin layer of polymeric material that adheres to the surface of food and that can be formulated based on edible biopolymers, mainly carbohydrates [13,14]. The application of these elements in meat products favors moisture retention, reduces the rate of deterioration reactions and the contamination and growth of microorganisms, and can even serve as a vehicle for the release of active compounds that incorporate additional preservative characteristics [14,15,16]. The formation of a uniform edible layer on the surface reduces the loss of components by limiting the transfer of gases including water vapor, as well as liquids [17]. To improve the permeability properties, structural integrity, and appearance of the coated products, the addition of plasticizers or additional constituents can be necessary in the forming solution [13,14]. One of the main challenges in meat matrices is the need to preserve quality properties and extend shelf life without altering or negatively impacting the sensory characteristics desired by the consumer [15,18]. In the development of new packaging systems for this type of product, it should also be seriously considered the package can contribute to the inactivation and/or reduction of the growth and activity of the microflora that can potentially cause deterioration or harm to the consumer [19,20]. Among the spoilage-causing microorganisms in chicken meat, *Pseudomonas* spp. are of greater concern. These psychrotrophic bacteria are active at refrigeration conditions and pH between 6.5 to 8.0. At these conditions, they generate proteinases that hydrolyze chicken protein and cause spoilage [8,21]. In chicken meat, spoilage bacteria such as *Pseudomonas* spp. use glucose from meat as a nutrient and once depleted, they can use most of the amino acids present, achieving a fast doubling time under cold storage, between 7.6 and 8.2 h to double the population, without being affected by the normal chicken pH. The effect of this is the appearance of green colorations, accompanied by rancidity of the fat fraction, firmness loss, and gelatinous appearances [22]. To counteract the deterioration caused by this type of microorganisms, several successful studies have been carried out with edible coatings with added antimicrobial activity, which are promising and considerably extend the shelf life of the product [19,23,24,25]. Khare et al. [19] used cinnamon oil in the edible coating to increase the shelf life of chicken breast in chilled storage, while Nouri and Shahbazi [24] used essential oils from *Ziziphora clinopodioides* and *Mentha spicata* with good results in inhibiting bacterial activity.

In order to develop a satisfactory edible coating, it is required that the used antimicrobial additives have high inhibition activity at low concentrations to avoid causing detriment in the sensory food properties and that these additives do not react with its constituent compounds. Likewise, it is required that the coating itself must be adapted to the product, preserving its quality characteristics as best as possible. With these considerations, the aims of this study were firstly, to select a suitable material as an edible coating for chicken breast fillets between guar gum and isolated soy protein, and then, secondly, to evaluate the effect of an edible coating based on guar gum added with plasticizers and natural antimicrobial agents on the physical, sensory, and microbiological characteristics of fresh chicken breast fillets stored under refrigerated conditions.

## 2. Materials and Methods

### 2.1. Meat Samples

Fresh chicken breast fillets were purchased from a commercial establishment in Bogotá, Colombia from birds slaughtered the previous day and then transported at 4 °C for testing. At the time of testing, the samples were washed with plenty of fresh water and a sodium hypochlorite solution 100 ppm, and then the surface water was removed to perform the coating and refrigerated storage trials. This process was made to remove dirt, reduce the initial microbial load, and improve adhesion and uniform coating formation.

### 2.2. Coating-Forming Compounds and Additives

The reagents used in the preparation of the coatings were USP (United States Pharmacopeia) grade. Oregano essential oil was obtained from dōTERRA^®^ (Pleasant Grove, UT, USA). Nisin (2.5% with a balance of sodium chloride from *Lactococcus lactis*) and polysorbate 80 were purchased from Sigma-Aldrich Co. (St Louis, MO, USA). Guar gum was supplied from GRINDSTED^®^ (DuPont—Danisco, Copenhagen, Denmark) and sorbitol was purchased from Merck KGaA (Darmstadt, Germany). The isolated soy protein (>99%) was purchased from Cimpa^®^ S.A.S. (Bogotá, Colombia).

### 2.3. Coating Formulation Selection

#### 2.3.1. Preliminary Concentration Tests

To define the coating-forming formulations of Table 1, several preliminary formulations between hydrocolloids and plasticizers were made. Concentrations between 1% y 5% (*w/v*) were tested for isolated soy protein and between 0.1% and 0.5% for guar gum. As for the plasticizers, these were tested in combinations of glycerol and sorbitol, between 0% and 5% (*w/v*).

#### 2.3.2. GG and ISP Coating Preparation

From preliminary concentration tests, four formulations for edible coatings were prepared from guar gum and isolated soy protein. For the preparation of the coatings, plasticizers (sorbitol and glycerol) and an emulsifier (polysorbate 80) were also added in the previously tested proportions indicated in Table 1. To obtain the coating-forming solutions, each hydrocolloid was diluted or dispersed in distilled water at 6 °C with constant stirring at 250 rpm. At the same time, an emulsion was prepared with the plasticizers and the emulsifier also in distilled water at 20 °C. After 10 min the stirring rate was increased up to 450 rpm and the solution was incorporated into the emulsion. Then, the temperature was increased up to 50 °C, keeping it constant for 20 min. Once the coating-forming solution was homogenized, it was placed in pre-sterilized glass containers and stored for 24 h at 5 °C to allow degassing. After this, test coatings were obtained for the four formulations indicated in Table 1 by the casting method. Then, 20 mL samples of the coating-forming solution were placed in non-stick molds made of Teflon^®^ with 4 cm in diameter. For each formulation in Table 1, 10 replicas were prepared which were dried at 60 °C for 24 h until obtaining edibles films. After drying, the films were detached from the mold and were then packed in polyamide sealable bags at 10 °C until characterization. The evaluated characteristics were thickness, transparency, color, and water activity. From the results obtained, a suitable formulation for the incorporation of antimicrobial compounds and coating tests in breast fillets was selected. The most successful formulation was later named as EC and used for the coating of the breast fillets samples.

Thickness was measured at five different points on each film (reporting the mean value) by using a Mitutoyo Digimatic^®^ micrometer, Model 293–240 (Mitutoyo, Kanagawa, Japan). To determine transparency in the films, samples of 2 cm × 1 cm rectangles were prepared with an aluminum mold, which were placed in adaptable plastic cells and taken to a GENESYS™ 10S UV-Vis Spectrophotometer (Thermo Fischer Scientific, Waltham, MA, USA) to measure absorbance and transmittance of each sample at a wavelength of 600 nm. Transparency was calculated using Equation (1) from Sánchez-Aldana et al. [26] as follows:

(1) $$\mathrm{Transparency}\;=\;\frac{A_{600}}{l}=\;\frac{-log\;T_{600}}{l},$$

where *A_600_* is the absorbance at 600 nm, *T_600_* is transmittance, and *l* is the sample thickness.

Color on the samples was determined according to the methodology described by Rhim et al., 2002 [27]. Color three-dimensional coordinates were determined by using a Minolta CT-400 colorimeter (Minolta Camera Co., Osaka, Japan) in coordinates L*, a*, and b* of the CIELAB color space, considering ‘Daylight 65’ as the standard illuminant with a 2° standard observer and lighting area of 8 mm. A white calibration plate (L* = 95.17 a* = −1.54 b* = 3.3) was used as a reference. Likewise, the total color difference (ΔE) was calculated relative to a cast polypropylene blank of 0.025 mm (Rediplast, Bogotá, Colombia) with Equation (2) [26]:

(2) $$∆E=\sqrt{{\left(L-L^{*}\right)}^{2}+{\left(a-a^{*}\right)}^{2}+{\left(b-b^{*}\right)}^{2}}.$$

The yellowness index (YI) of the films was determined by Equation (3) [26] from the L* and b* coordinates:

(3) $$\mathrm{YI}=142.86\frac{b^{*}}{L^{*}}.$$

Water activity (a_w_) was determined from film samples of approximately 0.5 cm^2^ for each formulation (Table 1) which were analyzed in a ROTRONIC^®^ HC2-AW water activity meter EC (Rotronic AG, Bassersdorf, Switzerland).

### 2.4. Antimicrobial Effect of Nisin and Oregano Essential Oil

In preliminary tests (unpublished), the maximum concentration of antimicrobial to be incorporated in the biopolymer-based EC solution was determined with 8 trained panelists, being it which did not affect the organoleptic characteristics of chicken breast fillets after cooking. Once the concentrations of nisin and oregano essential oil to be used were defined, 1% (*w/v*) and 0.05% (*w/v*) respectively, the individual and combined antimicrobial effect of the compounds were evaluated following the ISO 13720:2010 standard to determine the growth of microorganisms. Controlled in vitro inoculation of *Pseudomonas* spp. (strains available in the Laboratory of Microbiology, Institute of Biotechnology, National University of Colombia, Bogotá) was carried out in King’s A agar (Sigma-Aldrich Co., St Louis, MO, USA) with initial concentration <100 CFU/g, adding 0.1 cm^3^ of coating-forming solution (see below in Section 2.5.) with the antimicrobial concentration according to the treatment to be evaluated: control without compounds (T1), nisin and oregano essential oil (T2), oregano essential oil-only (T3), and nisin-only (T4). Incubation was performed at 25 ± 1 °C for 44 h. After that, samples were stored at 4 ± 1 °C until reaching a maximum growth of 10^8^ CFU/g [28,29]. This microorganism concentration was considered as the end of the food shelf life. All tests were performed in triplicate. From the observed results, the best antimicrobial treatment was chosen to carry out refrigerated storage tests with coated breast fillets.

### 2.5. Preparation of the Antimicrobial Coating Solution

The formulation selected after evaluating the thickness, transparency, color, and activity of the water was combined with the antimicrobial compounds in the highest concentrations previously tested that did not affect the sensory characteristics of the fillets. Hereinafter, this new combination was named EC. This new formulation was made in the solution with the most satisfactory hydrocolloid prepared in distilled water at 6 °C (0.4% *w/v*), with constant stirring at 250 rpm until a uniform mixture was obtained and then nisin was added (1.0% *w/v*) keeping steady stirring. The temperature was then increased up to 50 °C until the gelatinization of the mixture was reached. In parallel, an emulsion was prepared with sorbitol as plasticizer (1% *w/v*), polysorbate 80 as an emulsifier (0.1% *w/v*), and oregano essential oil (0.05% *w/v*) was added. The emulsion was kept at 50 °C and with stirring at 250 rpm for 15 min. Subsequently, the emulsion was incorporated into the hydrocolloid solution maintaining a constant temperature and increasing the stirring at 450 rpm for 20 min. Once the coating-forming solution was homogenized, it was placed in pre-sterilized glass containers and stored for 24 h at 5 °C to allow degassing.

### 2.6. Edible Coatings Application and Storage Tests

First, 200 ± 10 g of chicken breast fillets were coated by the dipping method for 10 s with the coating-forming solution EC, prepared as described above, draining excess of the coating solution for 40 s. The control samples (without coating) were immersed in distilled water for the same time as the coated ones. Once this was done, the coated samples and the control samples were placed in metal trays at 10 °C and 70% relative humidity for 1 h to let the coating dry. Then, the samples were stored at 4 ± 2 °C for 16 days, measuring the change in different physicochemical, microbiological, and sensory properties every two days until the end of the test. All measurements were done in triplicate, reporting the mean value of each measured property.

### 2.7. Measured Quality Properties

The following properties were evaluated for the samples of coated and control breast fillets:

The samples weight loss (WL) was determined by measuring the fillets weight on an Ohaus PA-3102 analytical balance (OHAUS Corp. Pine Brook, NJ, USA) with an accuracy of ± 0.01 mg and along the storage time, calculating the percentage of weight lost as follows Equation (4):

(4) $$\mathrm{WL}\%=\frac{\left(W_{\mathrm{ini}}-W_{t}\right)}{W_{\mathrm{ini}}}\times 100,$$

where W_ini_ is the initial weight and W_t_ is the sample weight at day t. The firmness associated with hardness was determined by taking sample pieces of a circular shape (by using a cylindrical punch) with 3 cm diameter and 3 cm long, which were analyzed in a Brookfield CT3 texturometer using a 2 mm rectangular steel probe. The compression test was performed with these parameters: trigger force 0.15 N, distance 5 mm, test speed 10 mm s^−1^. The color of the samples was determined with the Minolta colorimeter by making measurements at three points on the surface of each sample and taking the average value of the measurements. The color was reported in the color coordinates L*, a*, and b* of the CIELAB color space considering ‘Daylight 65′ as the standard illuminant. For the pH determination, approx. 5 g of each sample were taken and then macerated and mixed with 50 mL of distilled water with stirring for 5 min. Then, the pH was measured by introducing the probe of a Hanna HI4522 bench meter (Hanna Instruments, Woonsocket, RI, USA) into the mixture with steady stirring.

Sensory tests were carried out after cooking the breast fillets. For reducing a size bias, the coated and control fillets were cut into pieces of approximately 2.5 ± 0.1 cm^3^ use of a cylindrical punch and cooked on a hydrated griddle with 1 cm^3^ of vegetable oil for 3 min on each side until the internal temperature was 70 °C and adding 4 mg of salt per g of sample; these conditions were adapted from Ramirez [30]. The samples were evaluated by 8 semi-trained panelists using an attribute test with a structured scale. The overall appearance, color, odor, flavor, and texture attributes of the breast fillets were evaluated on a hedonic scale from 1 up to 5, with 1 being the unwanted characteristics and 5 being the suitable and favorable characteristics of the chicken breast fillets following the ISO-2859-1:1999 standard.

### 2.8. Statistical Analysis

To evaluate the effect of the treatments for the tests described in Section 2.3, Section 2.4, Section 2.5 and Section 2.6 (coating formulation, antimicrobial effect, and storage with the coated chicken fillets), a one-way analysis of variance was performed in each case with a significance level of 95% (α = 0.05) for the means (±standard deviation) of the measured properties. Significant differences between means of each property for the different treatments in each test were determined from Tukey’s HSD test. Statistical analyzes were performed by using the Statgraphics Centurion 18 software (Statgraphics Technologies, Inc. The Plains, VA, USA).

## 3. Results and Discussion

### 3.1. Formulation of the Coating-Forming Solution

In the preliminary concentration tests, it was observed that the characteristics of the coatings made with formulations with guar gum, and that included plasticizers in a percentage higher than 1%, were not favorable since it showed an excess of adhesiveness and moisture retention. For this reason, the sorbitol/glycerol concentration was limited up to 1% (*w/v*) in the formulations described in Table 1. For low concentrations of plasticizer (<1% *w/v*) such as that prepared for formulation F4, the films obtained presented a slightly brittle and opaque appearance compared to those obtained from formulation F3, showing a lower balance between the components. It can be attributed in this case to the lower proportion of plasticizers included and to the effect of the combination of sorbitol and glycerol, which had low synergy in F4. Consequently, the use of only 1% sorbitol in F3 was more favorable since it presented better elasticity and gloss.

Regarding the coating-forming component, the addition of 0.4% *w/v* of guar gum allowed the formation of transparent and glossy films with moderate elasticity, these characteristics being acceptable and suitable. Plasticizers, being hydrophilic liquids, share hydrogen bonds with the coating network, increase the free volume, and consequently promote the mechanical stability and flexibility of the coating, especially with the inclusion of only sorbitol for the F3 formulation [13]. For the films formed from isolated soy protein, a different behavior was observed for the GG films, since concentrations higher than 4% *w/v* of plasticizers were required to obtain continuous and low brittle films. Thickness obtained was as expected for an edible coating; in the GG-based formulations (Table 2), it was similar to those developed by Ayala et al. [31] based on xanthan gum, which ranges between 0.1 and 0.5 mm, or the films obtained by Sánchez-Aldana et al. [26] based on pectin (0.15–0.25 mm). These results are due, on the one hand, to the higher intermolecular forces that the GG chains possess, which allows them to constitute a more homogeneous and elastic film compared to the ISP ones. On the other hand, both gum and protein have a high affinity for water and form highly viscous solutions due to the extensive molecular branching they possess, trapping large amounts of solvent with strong interaction with each other, resulting in more humid, thicker, and opaque films in greater proportion for ISP [32,33]. It was observed that the lightness and transparency of the films obtained are related to a greater extent with the type of film-forming hydrocolloid used, due to its natural characteristics. ISP films (F1 and F2) had less transparency and lightness (L*) compared to GG formulations (F3 and F4). On the other hand, it was observed that the concentration, type, and combination of plasticizers also impact these attributes. For example, lightness was proportionally related to the concentration of plasticizers, since it was evidenced that the transparency values in GG films decrease when combining them. The total color difference (ΔE) between the ISP films and the PP blank film was higher (34.00–42.59) compared to the GG films (3.83–11.72). This may be due that for guar gum, a more homogeneous dispersion was obtained in the film-forming solution with the plasticizers used than in the case of soy protein. The films obtained from ISP in the experiments had a higher yellowness index and they were more opaque (lower L*) than those developed by Zuo et al. [34] based on zein and corn starch, and behaved in a similar way to those obtained by Mehyar et al. [35] based on whey protein, pea starch, and carnauba wax and those developed by Sharma and Singh [36] based on ISP and sesame protein. The transparency values in the guar gum films decreased with the addition of plasticizers greater than 0.5% (*w/v*). For these films, similar lightness and transparency values were obtained than those reported in other works of films based on pea starch and GG [12] and xanthan gum [37]. The mean value of a_w_ obtained in the experiments was 0.51 ± 0.05, without observing significant differences between them. These levels of water activity are not sufficient for microbial growth, although certain microorganisms such as osmophilic yeast and bacteria could survive for long periods at these conditions.

### 3.2. Antimicrobial Effect of Nisin and Oregano Essential Oil

**Figure 1:**
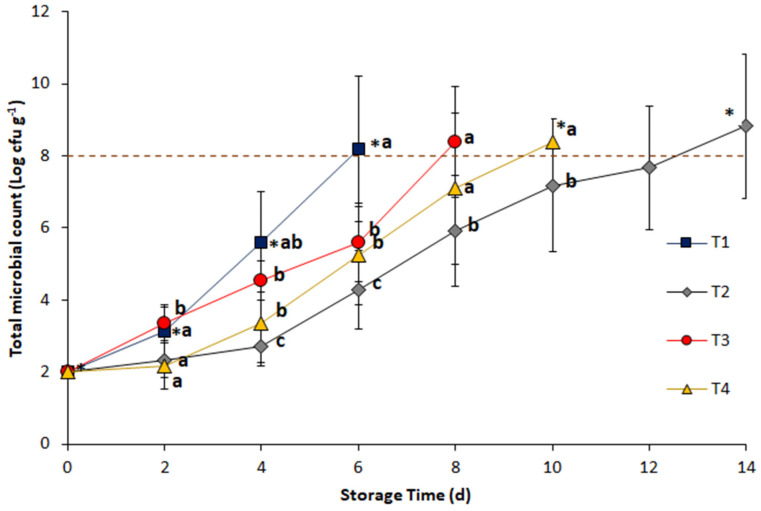
In vitro growth of *Pseudomonas spp.* under different antimicrobial treatments at 4 °C. T1: control; T2: 1% nisin + 0.05% oregano essential oil; T3: 0.05% oregano essential oil; T4: 1% nisin. Different letters indicate significant differences between treatments for the same sampling day. The asterisk indicates significant differences between consecutive measurement days for the same treatment according to Tukey’s HSD test (*p* ≤ 0.05). Standard deviation included (*n* = 3).

Preliminarily, the maximum concentration of nisin and oregano essential oil to be used were defined according to taste and overall acceptability, established through a structured scale sensory test. It was found that the maximum acceptable concentrations were 1%(*w/v*) for nisin and 0.05% (*w/v*) for oregano essential oil. After determining the maximum acceptable levels, the microbiological tests showed that in all cases, there was a sustained increase in microbial growth as shown in Figure 1. However, there were significant differences with slower microbial growth rates for T2 (0.05% *w/v* oregano oil + 1% *w/v* nisin) compared to T1 (control), T3 (0.05% *w/v* oregano oil), and T4 (1% *w/v* nisin). These differences were significant in the proliferation of *Pseudomonas spp*. from day 4. Regarding the T1 treatment (control), it showed a rapid increase in colonies, being significantly different from the other treatments from the second day of the test and also showing significant differences in the number of colonies present on each sampling day. On the other hand, there are no statistically significant differences between treatments T3 and T4, although treatment T3 (0.05% *w/v* oregano essential oil) reached the established limit of colony-forming units more quickly with respect to T4 (1% *w/v* nisin). This could be due to the higher concentration of nisin used for this study since previous works [38,39] have established the lower bactericidal activity of nisin against Gram-negative bacteria, although as shown in Figure 1, an antimicrobial potential is remaining, and also to the fact that the nisin used came with a balance of sodium chloride which could induce permeabilization of the cytoplasmic membrane in the microorganisms [40]. The results obtained for the essential oil of oregano are similar to those obtained by Govaris et al. [38] and da Silva et al. [41] who used it in higher concentrations (0.6% and 0.2% respectively). Likewise, and in a similar way to that obtained for the T2 treatment, Govaris et al. [38] controlled the growth of *Salmonella* spp. using essential oil of oregano (0.6% *w/v*) and nisin (500–1000 IU/g) combined in minced sheep meat. The results obtained showed the additive antimicrobial effect that was established between oregano essential oil and nisin, which was more efficient than the effect obtained separately for the antimicrobial compound. This additive effect was also observed by Natrajan and Sheldon [42] with nisin and citric acid applied in polymeric packages to preserve fresh broiler skin and by Sotoudeh et al. [43] with nisin and chitosan coatings for chicken breast fillets reducing the growth of *Salmonella* and *Staphylococcus aureus*.

### 3.3. Refrigerated Storage of Chicken Breast Fillets

**Figure 2:**
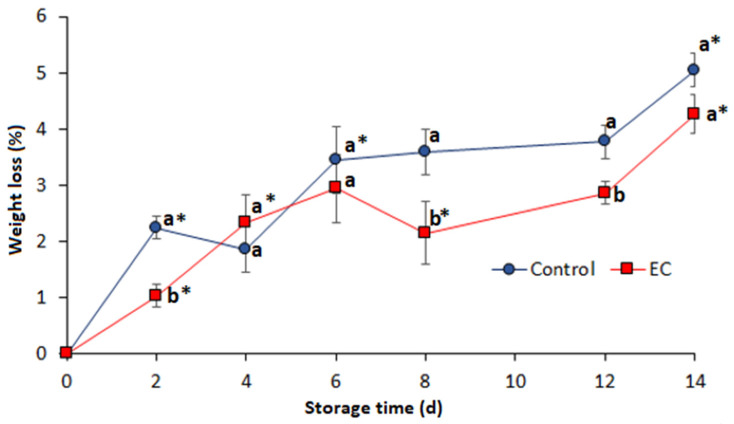
Weight loss (%) of chicken breast fillets with and without the antimicrobial coating stored at 4 °C. Different letters indicate significant differences between treatments for the same sampling day. The asterisk indicates significant differences between consecutive measurement days for the same treatment according to Tukey’s HSD test (*p* ≤ 0.05). Standard deviation included (*n* = 3).

In accordance with the properties described above and the better characteristics of the coating prepared from formulation F3 (0.4% guar gum + 1% sorbitol *w/v*), it was selected as EC, adding the antimicrobial compounds (1% nisin + 0.05% oregano essential oil *w/v*) and conducting the preservation test of the breast fillets. Weight loss during the refrigeration time showed significant differences (*p* ≤ 0.05) between treatments and between days of storage, as seen in Figure 2. Control fillets (uncoated) had a higher weight loss during the 14 days of storage.

In general, a continuous increase in weight loss was evidenced in both samples. Significant differences were found between treatments on the second day of storage and between the eighth and twelfth day, where the uncoated samples (control) had a greater weight loss. On day 14 of storage, there were no significant differences, but a higher weight loss was observed for both the control sample and the coated sample (5.1 ± 0.38% and 4.3 ± 0.44% respectively). The two treatments had significant losses on the second and fourteenth days of storage. The observed behavior can be attributed to the water retention capacity and the barrier effect of the GG coating that delays the moisture loss by evaporation due to the water activity differential between the samples and the surrounding atmosphere [25,44]. However, once the coating is saturated with moisture from the chicken sample, the cohesion of the GG molecules becomes lower and the water-holding capacity of the coating decreases, the permeability increases, therefore the weight loss of the breast fillets becomes higher as storage progresses [45]. This is partially compensated by the presence of plasticizers such as sorbitol, which increases the elongation and retention capacity of the water lost from the sample by the coating [46]. Nonetheless, the high loss of water from the chicken breast ends up exceeding the capacity of the coating. In meat foods such as chicken breast, the rate of moisture loss is high and its water-holding capacity decreases rapidly as proteins in muscle fibers break down [22,47].

The pH measurements showed significant differences between the treatments on days 12 and 14 of storage as shown in Table 3. Specifically, in time, there were significant changes in pH between the first 2 days and the remaining storage time, where a decrease in pH begins both in the fillets with coating EC (0.4% GG + 1% nisin + 0.05% essential oil of oregano) as in the control (without coating), said decrease being statistically greater in the fillets without coating. The mean pH value was 5.97 ± 0.09 for the control and 6.11 ± 0.10 for the coated fillets. However, although the coating exerted an effect on this parameter, these variations may also be due to the amount of glycogen contained in the muscle that is converted to lactic acid when oxygen supply ceases. While the control samples had a sustained decrease in pH with a value of 5.75 for day 14 of storage. For breast meat stored in refrigeration, steady pH values between 5.8–6.2 or a slight decrease in pH may be expected due to the formation of carbonic acid from the CO_2_ produced from the product by spoilage-causing microorganisms [48,49]. These results are like those obtained for the uncoated samples as shown in Table 3. On the other hand, in the case of samples of coated breast, constant pH values or a slight increase up to 6.5–6.8 have generally been observed, which agrees with the measured values for the coated samples (Table 3). Khare et al. [19] obtained a slight increase in pH for chicken fillets coated with carrageenan, citric acid, and cinnamon oil, while Giteru et al. [50] showed that the initial pH in fillets coated with kaphyrin with the addition of citral and quercetin was 5.95 and reached a maximum of 6.20 after 4 days of refrigerated storage. These results are similar to those obtained by Liang et al. [51] when applying an edible gelatin-coating added with nisin, EDTA, and potassium sorbate, and to those reported by Wang et al. [52] in packaging with modified atmospheres and application of cold plasma to breast fillets. In other cases, the acidic composition of the film-forming solution can result in pH reductions. In chitosan-coated chicken samples, a decrease in pH to 4.93 was reported after 12 days of refrigerated storage [52].

The pH can be linked to the color variations obtained in chicken breast fillets [22]. In Table 3, it is observed that lightness (L*) of the coated samples was similar to the control samples with values between 52–56 during the 14 days of evaluation without a clear trend of decrease or increase throughout the storage. The measured values were similar to those reported for chicken breast in other studies with average lightness values of 51–53 [53,54,55,56]. This similarity in L* for both coated and uncoated samples can be attributed to the high transparency of the GG coating formulation. These results were different from those obtained by Giteru et al. [50] with coatings of kafirin-based films incorporating citral, and quercetin and Ripoll et al. [6] with active zinc oxide and silver nanocomposite packaging who reported an increase in the sample lightness obtaining values above 60. However, as shown before in Table 2, with the addition of nisin and oregano essential oil, it does not appear to have altered the transparency of the coatings formed and the retention of water in the film did not cause distortions in the reflection of light, therefore the L* values remained steady. For the a* coordinate, a decrease was observed in the fillets evaluated, this being greater for the control samples. Regarding the b* coordinate, irregular changes were observed in the values of the samples without establishing a clear trend with values between 6–11 throughout storage as shown in Table 3. Initially, the application of the edible coating resulted in a greater pink-yellow coloration compared to the control (higher values of a* and b*). However, from day 6 of storage, there is a decrease in a* towards a greenish coloration, accompanied by a decrease in yellow coloration (b*), which may be a consequence of microbial activity and spoliation [22]. This behavior coincides with that reported by Ripoll et al. [6] where the red and yellow tones increased significantly from 0 to 7 days, and then slowly decreased.

**Figure 3:**
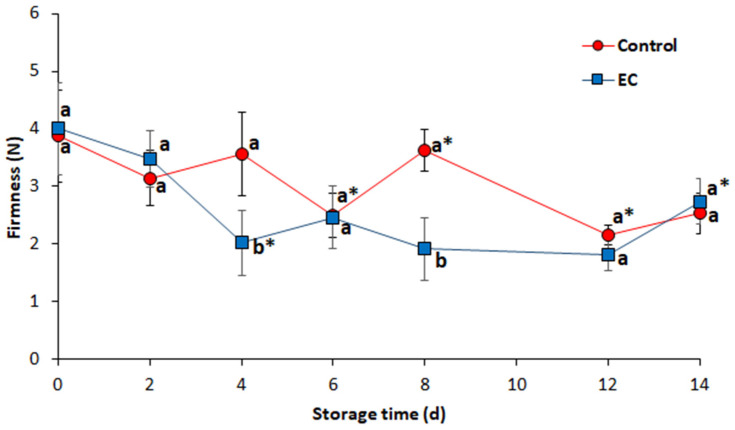
Firmness of chicken breast fillets with and without the antimicrobial coating stored at 4 °C. Different letters indicate significant differences between treatments for the same sampling day. The asterisk indicates significant differences between consecutive measurement days for the same treatment according to Tukey’s HSD test (*p* ≤ 0.05). Standard deviation included (*n* = 3).

Regarding the firmness of the samples, there was generally a decrease over the storage time for both the coated and uncoated fillets as shown in Figure 3. On day 6, the firmness of the coated and control samples decreased to 2.4–2.5 N, remaining at similar values until day 14, the significant differences between treatments were shown on the fourth and eighth day of storage, without a clear trend. However, the measured firmness values were lower for the coated fillets than for the control fillets (Figure 3). This can be attributed to a higher degradation of muscle fibers in the coated samples, perhaps due to the greater amount of moisture retained by the GG coating, which increased the development of protein degradation processes. In both cases, the firmness loss during the storage time could be associated with the hydrolysis of collagen, which produces the softening of tissues [22].

### 3.4. Sensory Evaluation of the Samples and Shelf life

**Figure 4:**
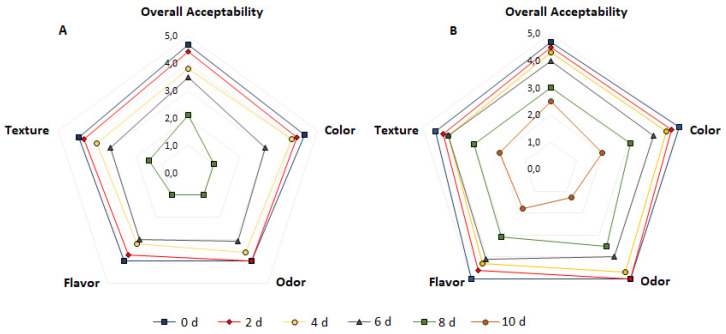
Sensory profile of chicken breast fillets without (**A**) and with (**B**) the EC antimicrobial coating stored at 4 °C.

**Figure 5:**
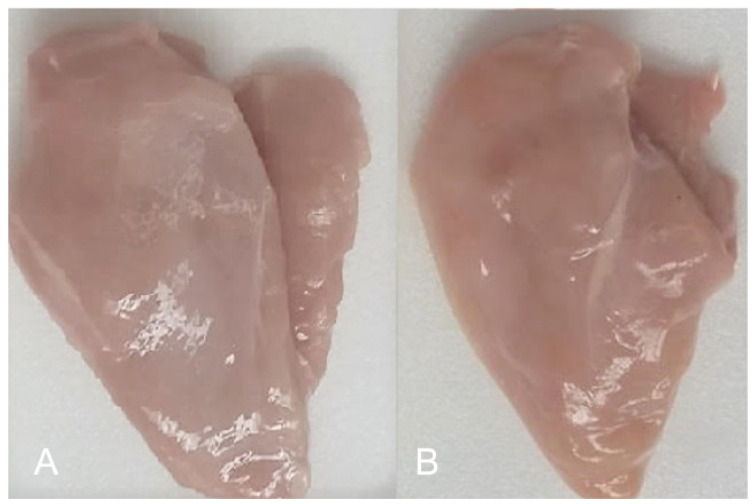
Visual comparison of chicken breast fillets with (**A**) and without (**B**) the EC antimicrobial.

Applying the coating to the fresh fillets resulted in residual oregano flavors and aromas that were well received by panelists and rated as pleasant, detectable, and long-lasting during chewing of chicken breast. The color was perceived as more homogeneous and golden compared to the uncoated samples, which can also be attributed to the adherence of the coatings in the fillet. As shown in Figure 4, the coated fillets were better rated for day zero than the control samples (approx. 4.8 vs. 4.2). This trend was preserved throughout the experiment. On the other hand, the application of the edible coating to the chicken breast fillets gave a higher gloss in the samples compared to the control and a slightly perceptible homogeneous layer on the surface of the product as shown in Figure 5. According to Gupta et al. [57], edible coatings are perceived as a variation in optical attributes such as color, gloss, and transparency, as well as generating changes in odor and external texture of the coated product. The colors obtained in the samples with the edible coating (EC) are usually transparent, but bright [58]. On the other hand, carbohydrate-based coated products achieve an attractive luster that is not sticky if it is applied to dry surfaces, and on the other hand, undesired condensation is avoided in the product packaging [59].

The evaluation of sensory characteristics was carried out until day 8 in the uncoated samples and until day 10 in the coated samples considering the perceived loss of quality in the physicochemical parameters and the microbiological results. Significant differences were observed from day 6 between the coated and control samples and between the subsequent evaluation days due to changes in the different sensory attributes evaluated (Figure 4). All the sensory attributes were reducing their score as storage progressed, although odor and color decreased more rapidly. In the case of uncoated fillets, the overall acceptability was reduced down to 2 for day 8, while color, odor, and flavor values were rated as 1 for this day. On the other hand, the addition of the edible coating delayed the processes of quality loss in the samples as shown in Figure 4. For day 8, the overall acceptability was scored as 3 and for day 10 with a value of 2.5 with similar values for the specific attributes. Fillet shelf life was estimated from sensory attributes and total acceptability. When the score reported by the panelists was less than 2.5 (acceptable), the product was considered to be no longer suitable for consumption and the end of its shelf life. Given this, the shelf life of the control samples was 6 days, while the shelf life of the samples coated with the GG antimicrobial film was 9 days. The results obtained are similar to other studies. Muñoz-Lescano [60] obtained improvements in the texture and color of breast fillets coated with a film based on guar gum with a shelf life of 13 days compared to 7 days for control. Eldaly et al. [53] obtained 12 days of shelf life for chicken breast fillets coated with a chitosan edible film and stored at 4 ± 1 °C compared to 3 days for control samples for that study. Regarding the evaluations conducted in this work, the application of the edible GG coating not only delayed deterioration but even (with the addition of active components such as oregano essential oil) improved some of the sensory characteristics of the samples evaluated such as flavor, odor, and color. In the future, it is possible to explore the improvement of certain characteristics of the edible coating, such as the addition of other components in combination with guar gum that allow a better moisture absorption without degrading its mechanical or permeability properties. This would result in less risk of deterioration by microorganisms and longer shelf life.

## 4. Conclusions

It was possible to obtain thinner coatings with higher lightness, transparency, and solubility by using guar gum coatings compared to those obtained with isolated soy protein. The use of sorbitol and glycerol as plasticizers improved the characteristics of the coatings obtained giving greater flexibility, uniformity, and adherence. Given the better observed characteristics for the coating formulated from guar gum, this was applied successfully for the preservation of the fresh chicken breast fillets improving the general appearance, color, odor, taste, and texture of the samples. It was evidenced that the oregano essential oil at 0.05% and nisin at 1% applied in the coatings added a bacteriostatic capacity against *Pseudomonas spp*., increasing shelf life satisfactorily. The deterioration of the coated samples occurred more slowly and to a lesser extent compared to the uncoated samples with lower weight loss and tissue softening and smaller changes in pH, color, a_w_, and also higher qualification of sensory attributes during storage under refrigerated conditions. The development of GG coatings with microbial agents such as oregano oil and nisin can be an interesting alternative for the best preservation of quality and shelf life of a fresh product such as chicken breast fillets.

## Figures and Tables

**Table 1:** Formulations for the preparation of guar gum and isolated soy protein edible films.

Compound (*w/v*%)	Formulation			
	F1	F2	F3	F4
Guar gum	-	-	0.4	0.4
Soy Isolate protein	4.0	4.0	-	-
Sorbitol	-	1.0	1.0	0.2
Glicerol	5.0	4.0	-	0.2
Polysorbate 80	0.1	0.1	0.1	0.1

**Table 2:** Characteristics of the films obtained for different formulations of guar gum and isolated soy protein.

Property	Formulation			
	F1	F2	F3	F4
Thickness (mm)	1.10 ± 0.05 a	1.13 ± 0.06 a	0.13 ± 0.06 b	0.07 ± 0.05 b
Transparency	0.76 ± 0.07 a	0.78 ± 0.01 a	0.88 ± 0.04 b	0.85 ± 0.06 c
L*	57.15 ±0.91 a	68.24 ±6,93 b	91,66 ±0,30 c	84.17 ±4.81 c
∆E	42.59 ± 1.63 a	34.00 ± 3.31 b	3.83 ± 0.27 c	11.72 ± 4.40 d
YI	56.01 ± 6.97 a	47.93 ± 5.67 a	7.14 ± 0.34 b	11.65 ± 1.86 b
a_w_	0.53 ± 0.07 a	0.50 ± 0.07 a	0.47 ± 0.06 a	0.55 ± 0.01 a

Standard deviation included for *n* = 10. Values within the same row with different letters indicate a significant difference between treatments by the Tukey’s HSD test (*p* ≤ 0.05).

**Table 3:** Changes in pH and color coordinates of coated and control chicken breast fillets during storage at 5 °C.

Property		Storage Time (d)						
		0	2	4	6	8	12	14
pH	Control	6.31 ± 0.04^a^_A_	6.26 ± 0.1 ^a^_A_	5.92 ± 0.11 ^a^_B_	5.92 ± 0.13 ^a^_B_	5.93 ± 0.14 ^a^_B_	5.73 ± 0.08 ^a^_B_	5.75 ± 0.02 ^a^_B_
	EC	6.20 ± 0.12 ^a^_A_	6.32 ± 0.08 ^a^_A_	5.97 ± 0.07 ^a^_B_	5.97 ± 0.15 ^a^_B_	6.11 ± 0.11 ^a^_C_	6.01 ± 0.02 ^b^_B_	6.18 ± 0.12 ^b^_C_
a*	Control	0.59 ± 0.64 ^a^_AB_	1.70 ± 0.31 ^a^_A_	1.61 ± 0.41 ^a^_A_	−4.04 ± 0.14 ^a^_B_	−3.65 ± 0.47 ^a^_B_	−0.99 ± 0.22 ^a^_C_	−4.01 ± 1.45 ^a^_B_
	EC	1.85 ± 0.95 ^a^_AB_	1.74 ± 0.72 ^a^_AB_	2.20 ± 0.68 ^b^_A_	−3.19 ± 0.74 ^a^_B_	−1.30 ± 0.26 ^b^_B_	−2.84 ± 3.01 ^a^_AB_	−1.63 ± 0.47 ^b^_AB_
b*	Control	9.80 ± 1.48 ^a^_AB_	7.31 ± 2.47 ^a^_AB_	6.84 ± 1.43 ^a^_AB_	9.19 ± 2.08 ^a^_AB_	6.39 ± 1.95 ^a^_AB_	6.69 ± 1.04 ^a^_A_	9.91 ± 2.59 ^a^_B_
	EC	10.67 ± 0.83 ^a^_A_	8.10 ± 1.09 ^a^_A_	7.74 ± 0.93 ^a^_A_	10.27 ± 1.65 ^a^_A_	6.69 ± 0.88 ^a^_A_	8.82 ± 1.24 ^a^_A_	8.06 ± 1.86 ^a^_A_
L*	Control	54.09 ± 3.61 ^a^_A_	55.62 ± 3.68 ^a^_A_	52.53 ± 3.30 ^a^_A_	55.29 ± 0.73 ^a^_A_	55.16 ± 1.65 ^a^_A_	54.86 ± 4.33 ^a^_A_	54.88 ± 4.95 ^a^_A_
	EC	55.88 ± 0.86 ^a^_A_	53.09 ± 3.01 ^a^_A_	55.19 ± 0.88 ^a^_A_	55.78 ± 1.97 ^a^_A_	53.70 ± 3.06 ^a^_A_	54.93 ± 2.76 ^a^_A_	54.50 ± 3.08 ^a^_A_

Standard deviation included for *n* = 3. In columns, within treatments, means with the same lowercase superscripted letter are not significantly different. In rows, for each treatment and within storage days, means with the same capital subscripted letter are not significantly different, by the Tukey’s HSD test (*p* ≤ 0.05).
